# The application of an oxygen mask, without supplemental oxygen, improved oxygenation in patients with severe COVID-19 already treated with high-flow nasal cannula

**DOI:** 10.1186/s13054-021-03738-8

**Published:** 2021-08-28

**Authors:** Besarta Dogani, Fredrik Månsson, Fredrik Resman, Hannes Hartman, Johan Tham, Gustav Torisson

**Affiliations:** 1 Department of Internal Medicine, Skåne University Hospital, Malmö, Sweden; 2 Department of Infectious Diseases, Skåne University Hospital, Ruth Lundskogs Gata 3, 20502 Malmö, Sweden; 3 Clinical Infection Medicine, Department of Translational Medicine, Lund University, Malmö, Sweden; 4 Department of Clinical Sciences Malmö, Lund University, Skåne University Hospital, Malmö, Sweden

*Trial registration* ClinicalTrials, NCT04794400 Registered 12 March 2021—Retrospectively registered, https://clinicaltrials.gov/ct2/show/NCT04794400

Hypoxemia is the clinical hallmark of severe COVID-19 infection, and guidelines suggest using high-flow nasal cannula (HFNC) when conventional oxygen therapy fails [1, 2]. In late 2020, we observed that oxygenation could be improved in some patients by applying a mask (e.g. a nebulisation mask or simple oxygen mask) to ongoing HFNC. This procedure has quickly become a clinical routine at our hospital, and in this study, we aim to assess its effect.

The study was performed at Skåne university hospital in Malmö, Sweden. Eligibility criteria were (1) COVID-19 infection, (2) HFNC treatment, and (3) estimated PaO2/FiO2 ratio of ≤ 13 kPa (~ 97.5 mmHg). Baseline measurements, including arterial blood gases (ABG), were taken without mask. Then, a simple oxygen mask was applied over nose and mouth for 30 min, without supplemental oxygen, followed by another ABG. Patients maintained position and HFNC settings throughout the procedure, which was monitored by a study physician. After mask removal, SpO2 was recorded upon reaching steady state and participants could continue using the mask at their doctor’s discretion. The primary outcome was change in SaO2, with hypothesis testing through a paired t test. Secondary outcomes included changes in PaCO2, SpO2 and respiratory rate.

Eighteen patients were included, see Table 1. SaO2 (%) was higher in all patients after 30 min with mask than at baseline, mean difference: 5.1% (95%CI 3.0–7.2%), see Fig. 1a. There was a trend towards increased PaCO2, mean difference: 0.15 (95%CI − 0.03 to 0.34) KPa, see Fig. 1b. SpO2 increased with mask and decreased after mask removal, see Fig. 1c. Mean respiratory rate was 22.4 with mask, compared to 24.6 at baseline, mean difference: − 2.2, (95%CI − 0.2 to − 4.2).

**Table 1:** Patient and infection characteristics at the time of inclusion

	N = 18
Age	69 (61–75)
Male sex	13 (72%)
Smoking history	10 (56%)
Diabetes	3 (17%)
Hypertension	11 (61%)
Chronic pulmonary disease	4 (22%)
Immunosuppression	3 (17%)
Charlson index ≥ 2points	6 (33%)
Body mass index	
< 25	3 (17%)
25–30	8 (44%)
30–35	1 (6%)
35 +	6 (33%)
Infection characteristics	
Symptom duration, days	13 (10–14)
Respiratory rate/minute	24 (21–28)
Heart rate/minute	69.5 (64–87)
MAP, mmHg	90 (84–103)
PaO2, KPa/mmHg	8.1 (7.0–8.8) / 61 (53–66)
Estimated P/F ratio, KPa/mmHg	9.8 (8.4–10.5) / 74 (63–79)
c-Reactive protein, mg/L	74 (41–111)
Neutrofile/lymphocyte ratio	12 (7–21)
Procalcitonin, μg/L	0.2 (0.1–0.3)
Ferritin, μg/L	1085 (752–1683)
d-Dimer, mg/L	1.9 (0.8–3.6)
Troponin, ng/L	12 (9–21)
Pro-BNP, ng/L	466 (235–1197)
Creatinine, µmol/L	65 (59–73)
Treatment	
Betametasone	18 (100%)
LMWH	18 (100%)
Remdesivir	1 (6%)
Antibiotics	6 (33%)
HFNC flow, L/min	40 (40–40)
FiO2, %	82.5% (80–100)
Position (side/back/prone)	9/4/5
HFNC/NIV ceiling of care	4 (22%)

Data are presented as median (IQR) or count (%). For Charlson index, the updated version (Quan 2011) was used

MAP: mean arterial pressure; BNP: brain natriuretic peptide; LMWH: low-molecular weight heparin; HFNC: high-flow nasal cannula; FiO2: fraction of inspired oxygen; NIV: non-invasive ventilation

**Fig. 1:**
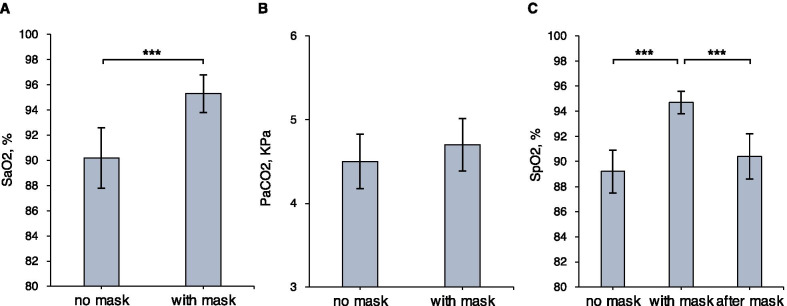
Outcome. Mean oxygen saturation from arterial blood (SaO2) at baseline and with mask (**a**). Mean partial pressure of carbon dioxide (PaCO2) at baseline and with mask (**b**). Mean peripheral saturation from pulsoximetry (SpO2) at baseline, with mask and after mask removal (**c**) error bars = 95% confidence interval of the mean. *** = *p* < .001 from two-sided paired *t* test. Paired nonparametric tests were also performed to test robustness, with a similar degree of statistical significance (*p* < .001)

Thus, this small study confirmed the observation that oxygenation improved when a mask was added to HFNC. PaCO2 increased slightly, possibly due to a lower respiratory rate, but without hypercapnia. No other side effects or complications were observed during this short-term study. The decline of SpO2 after mask removal suggested an intervention effect, although SpO2 did not fully reach baseline levels. The underlying mechanism was not studied, but we hypothesise that the mask could minimise entrainment of room air, especially when mouth-breathing.

Our HFNC device had a maximum flow rate of 40 L/min. However, the increase in SaO2 of 5% is in line with the 4% found in a study with a similar design but another HFNC device and a flow rate of 60 L/min [3]. Furthermore, this other study used a surgical mask, suggesting that the observed phenomenon is neither strictly mask- nor device-dependent. The study populations of these two small studies were quite similar though, and the generalisability of the results must be considered uncertain at this point.

Optimal intubation timing in COVID-19 is debated [4–6]. At our hospital, patients with severe hypoxemia have increasingly been managed for long periods on non-invasive respiratory support, including awake proning. In this context, the intermittent use of mask + HFNC (alternating with proning, during mobilisation, as a rescue in desaturation episodes, a bridge to intubation or a last resort for patients with ceiling of care) has filled a niche, being less demanding than NIV by face mask, while maintaining benefits of HFNC over conventional oxygen treatment. However, without experienced staff, rigorous monitoring and intubation protocols, adding a mask to HFNC could also delay intubation, putting the patient at risk.

In conclusion, further studies are needed regarding oxygen delivery in severe COVID-19. The results in this study suggest that the addition of a mask to HFNC could improve oxygenation in some patients in the short-term perspective. However, potential long-term risks, including those associated with delaying intubation, must be acknowledged.

## Declarations

### Availability of data and materials

Data are available on reasonable request to the corresponding author.
